# A relationship between a level of hemoglobin after delivery and exclusive breastfeeding initiation at a baby friendly hospital in Japan

**DOI:** 10.1186/s12199-017-0650-7

**Published:** 2017-04-20

**Authors:** Saki Horie, Kyoko Nomura, Shinichi Takenoshita, Junko Nakagawa, Michiko Kido, Mitsuhiro Sugimoto

**Affiliations:** 1 Teikyo University Graduate School of Public Health, Tokyo, Japan; 2 National Institute of Health and Nutrition, Tokyo, Japan; 3 Department of Hygiene and Public Health, School of Medicine, Teikyo University, 2-11-1 Kaga, Itabashi-ku, Zip 173-8605 Tokyo Japan; 4 Department of Obstetrics and Gynecology, Japanese Red Cross Medical Center, Tokyo, Japan; 5 Department of Obstetrics and Gynecology, Tohto Bunkyo Hospital, Tokyo, Japan

**Keywords:** Exclusive breastfeeding, Anemia, Hemoglobin, Underweight, Postpartum hemorrhage

## Abstract

**Background:**

The recent National Nutrition Survey of 2013 demonstrated that 16.7% of women in childbearing age are underweight, and 5.0–10.0% of these women manifested a Hemoglobin (Hb) level less than 11.0 g/dl. The purpose of this study was to investigate if such maternal nutritional status affects success of exclusive breastfeeding (EBF) practice.

**Methods:**

This cross-sectional study investigated 1532 dyads of mothers and infants with full-term singleton pregnancies delivered during 2011 at a perinatal center in Tokyo. Outcome is EBF initiation defined as the successful practice at discharge and 1 month after discharge. A logistic regression model was applied to investigate the impact of Hb levels (<9.0, 9.0–10.9, and ≥11.0 g/dl) measured within 2–3 days after delivery on successful EBF initiation adjusting for covariates including bleeding at delivery.

**Results:**

Mean age was 34 years, 23.0% were underweight and 63.0% were nulliparous. The success rate for EBF initiation at discharge and at 1 month after discharge was 72.7 and 63.0% for a Hb level <9.0 g/dl, 81.9 and 68.9% for a Hb level of 9.0–10.9 g/dl, and 85.7 and 75.9% for a Hb level ≥11.0 g/dl, respectively. A logistic regression model showed that risk factors of unsuccessful EBF practice at discharge and 1 month after discharge included lower level Hb categories (*P* < 0.001 and *P* < 0.001), postpartum hemorrhage > 500 ml (*P* = 0.089 and *P* = 0.011), maternal age (*P* < 0.001 and *P* < 0.001), nulliparity (*P* < 0.0001 and *P* < 0.001), pregnancy-induced hypertension (*P* = 0.002 and *P* = 0.012), gestational week (*P* = 0.006 and *P* = 0.002), Low Birth Weight (LBW) (*P* < 0.001 and *P* < 0.001), and immediate separation (*P* < 0.001 and *P* = 0.020). After adjusting for the covariates, compared with a Hb level ≥11.0 g/dl, a Hb level <9.0 g/dl was significantly associated with unsuccessful EBF initiation at discharge [odds ratio (OR): 2.15; 95% confidence interval (CI): 1.37–3.39] and at 1 month after discharge (OR: 1.63; 95% CI: 1.10–2.42), and a Hb level of 9.0–10.9 g/dl also was significant at 1 month after discharge (OR: 1.35; 95% CI: 1.04–1.75). Pre-pregnancy underweight was not associated with success of EBF practice both at hospital discharge and 1 month after discharge.

**Conclusion:**

Maternal severe anemia after delivery was associated with the risk of unsuccessful initiation of EBF even after adjusting for bleeding at delivery, suggesting the importance of dietary management especially in the later trimester.

## Background

The World Health Organization (WHO) recommends breastfeeding only breast milk without water or anything else until 6 months of age. They also recommend continuing breastfeeding even after 2 years of age with supplementation with the appropriate diet. Increasing the breastfeeding rate not only reduces children’s diarrheal diseases and respiratory infections but also leads to increased intelligence levels, obesity prevention, diabetes mellitus even during subsequent growth, and maternal breast and ovarian cancer.

In Japan, the rate of successful exclusive breastfeeding (EBF) practice at 1 month after delivery is 42.0% according to the National Infants Nutrition Survey of 2005 [1], which is very low compared to other countries, although more than 96.0% of those expectant mothers intended to breastfeed before delivery. Thus, it requires epidemiological studies conducted to elucidate factors associated with unsuccessful breastfeeding practice, and then a useful countermeasure should be immediately introduced for a improved health outcome of mothers and infants.

Among factors associated with unsuccessful EBF practice, poor maternal nutritional status is considered as one of the biggest public health concerns in Japan. The recent National Nutrition Survey of 2013 demonstrated that 16.7% of women in childbearing age are underweight, defined as a body mass index (BMI) <18.5 kg/m^2^: 21.5% in their 20’s, 17.6% in their 30’s and 11.0% in their 40’s are underweight. Such prevalence of being underweight may be brought by reduced caloric intake; the survey reported the caloric intake of women of child-bearing age (i.e., 20’s to 40’s) to be 1628–1665 kcal/day, a value far below the requirements established by the 2015 Dietary Reference Intakes for Japanese of 1950 kcal/day for women 18–29 years and 2000 kcal/day for women 30–49 years of age with moderate physical activity levels. Moreover, maternal underweight status is related to iron-deficiency anemia. According to the National Nutrition Survey of 2008, approximately 5.0% of women in their 20’s and 10.0% of women in their 30–40’s manifested Hemoglobin (Hb) level less than 11.0 g/dl. Hence, the purpose of this study was to investigate the impact of Hb level with or without pre-pregnancy BMI on successful initiation of EBF among Japanese women.

## Methods

In this cross-sectional study, we obtained an anonymous dataset of consecutive deliveries performed between January 2011 and December 2011, extracted from electronic medical records at a hospital in the Tokyo metropolitan area, which had the second largest number of deliveries in Japan. This hospital is also accredited as a baby-friendly hospital (BFH) in Tokyo. Items obtained from the medical records were Hb levels measured within 2–3 days after delivery (<9.0, 9.0–10.9, and ≥11.0 g/dl; anemia was defined according to WHO criteria i.e. Haemoglobin <11 gm/dl.), amount of bleeding at delivery, maternal age (years), parity (primipara or multipara), pre-pregnancy BMI (<18.5, 18.5–24.9, and ≥25.0 kg/m^2^), pregnancy-induced hypertension (PIH), delivery method (vaginal or Caesarean section), gestational week at delivery, low birthweight infant (<2500 g, LBW), and separation after delivery which indicates that a mother and her baby were separated due to clinical reasons and unable to breastfeed. Maternal hypertension was defined as high blood pressure (i.e., ≥140/90 mmHg) [2]. This study was approved by the ethics committee of Teikyo University School of Medicine, Tokyo, Japan (TU-COI 13–1592). The details are described elsewhere [3].

Our dataset primarily contained 1618 full-term dyads delivered at 37–42 gestational weeks after excluding miscarriage (*n* = 220), preterm birth (*n* = 253), multiple pregnancies (*n* = 81), and stillbirths (*n* = 10). For the purpose of our research hypothesis that focused on the impact of underweight on EBF initiation, we further excluded obese mothers with pre-pregnancy BMI ≥ 25.0 (*n* = 82), missing data on Hb levels (*n* = 2), and separation after delivery (*n* = 2). Finally, we used 1532 dyads for breastfeeding practice investigation at discharge and further excluded subjects with missing data on breastfeeding practice at 1 month (*n* = 24); we used 1508 dyads for breastfeeding investigation at 1 month.

The outcome of interest in the present study was successful EBF initiation which was defined as successful EBF practice within 24 h of hospital discharge and at 1 month after discharge. According to the World Health Organization/United Nations Children’s Fund definition, EBF practice is defined as when infants are fed only breast milk from his/her mother or wet nurse through breastfeeding or expressed breast milk, with no other liquids or solids given (except for drops or syrups containing nutritional supplements or medicine) [4]. Partial breastfeeding included infants who were breastfed in addition to artificial feeds, excluding those who were fed only formula.

An association between Hb category levels and explanatory variables were statistically assessed by a chi-square test or analysis of variance as appropriate. Categorical data such as prepregnancy BMI, nulliparity, pregnancy induced hypertension and so forth were assessed by using the chi-square test, and continuous variables such as maternal age, gestational weeks, bleeding amount at delivery and so forth were assessed by using an analysis of variance. A logistic regression model (i.e., EBF = 1 vs. partial breastfeeding or formula only = 0) were used to compute odds ratios (OR) for unsuccessful EBF practice, together with 95% confidence intervals (CI). Amount of bleeding at delivery was categorized into binary as postpartum obstetrical hemorrhage >500 ml or ≤500 ml. In multivariable analyses, covariates selected at *p* < 0.01 at univariate models was used while forcedly including both pre-pregnancy BMI and Hb levels for the purpose of our research hypothesis to see the effect of being underweight and anemia on successful EBF practice. All of the logistic regression analyses were performed at hospital discharge and at 1 month after discharge. Statistical interactions were performed by including the interaction terms of pre-pregnancy BMI and Hb category levels.

All of the data were analyzed using SAS version 9.4 for Windows (Cary, NC, USA). All of the CIs were at the 95% level, and statistical significance was set at a *P*-value of less than 0.05.

## Results

Table 1 shows the characteristics of participants according to Hb category levels. Average age of the mothers was 34 years, 48.0% were 35 years or older, and 63.0% were primiparous. A significant portion of the mothers in our study (63.0%) had anemia with a Hb level <11.0 g/dl; among these, 13.0% had severe anemia, defined as Hb level <9.0 g/dl. One-fourth of our subjects were underweight (23.0%), defined as BMI <18.5 kg/m^2^. The proportion of subjects with Hb level < 9.0 g/dl was similar between underweight mothers and normal weight mothers (13.4% vs. 13.4%; *P* = 0.987). The lowest Hb level category (<9.0 g/dl) was associated with nulliparity (*P* < 0.001), cesarean section (*P* < 0.001), PIH (*P* = 0.002), higher gestational weeks (*P* < 0.001), larger bleeding amount (*P* < 0.001), and birth weight of an infant (*P* < 0.001).

**Table 1:** Characteristics of participants according to Hb category levels (*n* = 1532)

	Hb < 9.0		9.0 ≤ Hb ≤ 11.0		12.0 ≤ Hb		P*
	*n* = 205		*n* = 762		*n* = 565		
	N	%	N	%	N	%	
Maternal age, years, mean ± SD	33.9	5.4	34.1	4.8	33.7	5.1	0.484
Pre-pregnancy BMI, kg/m^2^							0.925
< 18.5	48	23.4	175	23.0	135	23.9	
18.5–24.9	157	76.6	587	77.0	430	76.1	
Nulliparity	153	74.6	492	64.6	323	57.2	<0.001
Caesarean section	49	24.0	134	17.6	61	10.8	<0.001
Pregnancy Induced Hypertension	17	8.3	26	3.4	17	3.0	0.002
Gestational week at delivery, mean ± SD	39.5	1.1	39.4	1.1	39.2	1.1	<0.001
Bleeding amount at delivery, mean ± SD	907.6	473.5	519.1	291.4	366.8	254.2	<0.001
Birth weight of infant, mean ± SD	3127.8	345.1	3083.4	354.3	3029.1	352.0	<0.001
Immediate separation after delivery	21	10.2	51	6.7	34	6.0	0.117
Breastfeeding at discharge	149	72.7	624	81.9	484	85.7	<0.001
Breastfeeding at 1 month	126	63.0	518	68.9	422	75.9	<0.001

*Based on Chi-square test or Analysis of Variance

The success rates for EBF initiation at discharge were 72.7% for a Hb level <9.0 g/dl, 81.9% for a Hb level of 9.0–10.9 g/dl, and 85.7% for a Hb level ≥11.0 g/dl and decreased at 1 month after discharge down to 63.0, 68.9, and 75.9%, respectively. Table 2 shows the effects of the Hb level on unsuccessful EBF initiation at discharge. A logistic regression model showed that risk factors of unsuccessful EBF initiation at discharge included lower level of Hb categories (*P* < 0.001), higher maternal age (*P* < 0.001), nulliparity (*P* < 0.001), cesarean section (*P* = 0.003), pregnancy-induced hypertension (*P* = 0.002), gestational weeks (*P* = 0.006), postpartum obstetrical hemorrhage >500 ml (*P* = 0.089), LBW (*P* < 0.001), and immediate separation (*P* < 0.001). Multivariable model demonstrated that Hb levels <9.0 g/dl were significantly associated with unsuccessful EBF initiation at discharge (OR: 2.15; 95% CI: 1.37–3.39).

**Table 2:** Proportion and odds ratio (OR) with 95% confidence interval (CI) for mothers who did not initiate exclusive breastfeeding (EBF) at discharge (*N* = 1532)

	Logistic regression models					
	Crude OR (95% CI)	P*	Multivariate stepwise models			
			Adjusted OR	95% CI		P*
				Lower	Upper	
Maternal age	1.10 (1.07–1.13)	<0.001	1.12	1.09	1.16	<0.001
Prepregnancy BMI, kg/m^2^		0.722				0.810
< 18.5	0.95 (0.69–1.29)		1.04	0.77	1.46	
18.5–24.9	1		1			
Nulliparity	2.18 (1.61–2.94)	<0.001	2.38	1.72	3.30	<0.001
Caesarean section	1.63 (1.18–2.26)	0.003	1.04	0.71	1.52	0.832
Pregnancy Induced Hypertension	2.39 (1.37–4.15)	0.002	1.30	0.70	2.42	0.410
Gestational week at delivery	0.85 (0.75–0.95)	0.006	0.84	0.73	0.95	0.006
postpartum obstetrical hemorrhage >500 ml	1.26 (0.97–1.64)	0.089	0.91	0.66	1.25	0.545
Hemoglobin level		<0.001				0.004
< 9	2.25 (1.53–3.31)		2.15	1.37	3.39	
9–11	1.32 (0.98–1.78)		1.27	0.92	1.75	
12≤	1		1			
Birthweight of infant <2500 g	4.04 (2.51–6.53)	<0.001	2.72	1.58	4.67	<0.001
Immediate separation after delivery	2.80 (1.84–4.27)	<0.001	2.61	1.66	4.10	<0.001

*P for the category or trend p

Table 3 shows the effects of the Hb level on unsuccessful EBF practice 1 month after discharge. A logistic regression model showed that risk factors of unsuccessful EBF practice 1 month after discharge included lower level of Hb categories (*P* < 0.001), higher maternal age (*P* < 0.001), nulliparity (*P* < 0.001), cesarean section (*P* < 0.001), pregnancy-induced hypertension (*P* = 0.012), gestational week (*P* = 0.002), postpartum obstetrical hemorrhage >500 ml (*P* = 0.011), LBW (*P* < 0.001), and immediate separation (*P* = 0.020). Multivariable model demonstrated that a Hb level of <9.0 g/dl (OR: 1.63; 95% CI: 1.10–2.42) and 9.0–10.9 g/dl (OR: 1.35; 95% CI: 1.04–1.75) were significantly associated with unsuccessful EBF practice 1 month after discharge. Pre-pregnancy underweight status was not associated with success of EBF practice both at discharge and at 1 month after discharge.

**Table 3:** Proportion and odds ratio (OR) with 95% confidence interval (CI) for mothers who did not initiate exclusive breastfeeding (EBF) at 1 month (*N* = 1508)

	Logistic regression models					
	Crude OR (95% CI)	P*	Multivariate stepwise models			
			Adjusted OR	95% CI		P*
				Lower	Upper	
Maternal age	1.05 (1.03–1.08)	<0.001	1.06	1.03	1.08	<0.001
Prepregnancy BMI, kg/m2		0.442				0.941
<18.5	0.90 (0.69–1.18)		1.01	0.77	1.33	
18.5–24.9	1		1			
Nulliparity	1.66 (1.31–2.11)	<0.001	1.73	1.34	2.24	<0.001
Caesarean section	2.13 (1.61–2.83)	<0.001	1.66	1.22	2.26	0.001
Pregnancy induced hypertension	1.96 (1.16– 3.31)	0.012	1.29	0.74	2.27	0.373
Gestational week at delivery	0.85 (0.77–0.95)	0.002	0.84	0.75	0.94	0.002
postpartum obstetrical hemorrhage >500 ml	1.34 (1.07–1.68)	0.011	1.01	0.77	1.31	0.973
Hemoglobin level		<0.001				0.025
<9	1.85 (1.31–2.62)		1.63	1.10	2.42	
9–10.9	1.42 (1.11–1.82)		1.35	1.04	1.75	
11≤	1		1			
Birthweight of infant <2500 g	2.39 (1.49–3.85)	<0.001	1.63	0.97	2.74	0.063
Immediate separation after delivery	1.64 (1.08–2.48)	0.020	1.44	0.93	2.22	0.100

*P for the category or trend p

## Discussion

This study demonstrated that maternal anemia, defined as an Hb level <9.0 g/dl, was inversely associated with successful initiation of EBF at discharge and 1 month after discharge, and an Hb level of 9.0–10.9 g/dl was inversely associated with successful initiation of EBF at 1 month after discharge. On the other hand, being underweight defined as pre-pregnancy BMI <18.5, was not associated with success of EBF practice both at hospital discharge and 1 month after discharge.

In Japan, the rate of successful EBF practice at 1 month after delivery is 42.0% [1], although more than 96.0% of those expectant mothers intended to breastfeed before delivery. The recent National Nutrition Survey of 2013 demonstrated that 16.7% of women in childbearing age are underweight, and 5.0–10.0% of these women manifested Hb level of less than 11.0 g/dl. Our research hypothesis was that such maternal nutritional status may negatively affect EBF practice. However, we found that underweight status in women in the reproductive age was not associated with the risk for successful initiation of EBF. It may also reflect maternal pro-anemic status in later trimester because the association still remained even after adjusting for bleeding amount at delivery. The result of our present study may suggest that future studies should be conducted to investigate the Hb levels of mothers in the third trimester and its association with successful EBF adjusting for bleeding amount at delivery.

On the other hand, we found that the lowest Hb level <9.0 g/dl was inversely associated with successful EBF initiation. Because the level of Hb was measured 2 or 3 days after delivery, its low level may both reflect anemia due to massive bleeding at delivery and prolonged anemia carried over from the third trimester period. In this study, we found that the relationship between anemia and unsuccessful initiation of EBF remained significant even after adjusting for obstetrical bleeding. This may indicate maternal pro-anemic status in later trimester may also be associated with the success of EBF practice. In this regard, a future study should be conducted to investigate the Hb levels of mothers in the third trimester and its association with successful EBF practice.

Although there are very few studies that have investigated why a lower level of Hb is inversely associated with a successful EBF initiation, one possible mechanism of anemia on milk production is explained by the hypothalamic-pituitary-adrenal axis [5], which is central to the regulation of breastfeeding [6]. As evidenced by Sheehan’s syndrome, stress at delivery, including postpartum hemorrhage, impairs the anterior lobe pituitary, which may impair milk production via decreased levels of prolactin caused by hypopituitarism [7]. Although little is known about Sheehan’s syndrome, which is difficult to diagnose [8], a 20-year cohort study in Costa Rica [9] investigated 60 cases diagnosed with Sheehan’s syndrome. According to this report, the average time between the previous obstetric event and a diagnosis of Sheehan’s syndrome was 13 years, and the most frequent obstetric antecedents were a history of obstetric hemorrhage (82.0%), shock (47.0%), and blood transfusion (43.0%), and agalactia was observed in 67.0% of all reported cases. Other studies in France [10] and Turkey [11, 12] that investigated patients with Sheehan’s syndrome consistently reported that most of their patients had agalactia [10–12] and a massive postpartum hemorrhage event [10, 12]. Obstetrical hemorrhage, defined as a bleeding volume of 500 ml or more [13], is a leading cause of maternal deaths in Japan. Among our study participants, 39.0% experienced an obstetrical hemorrhage event, defined as a 500 ml or greater bleeding volume. Additionally, in the group with a Hb level <9.0 g/dl, 81.0% experienced an obstetrical hemorrhage event. Although we did not find a direct relationship between obstetrical hemorrhage and EBF practice and we have not measured prolactin levels in this study, our results suggest that mothers who experience critical postpartum hemorrhaging and failure to establish breastfeeding should be monitored for prolactin levels [14].

Several limitations of this study should be addressed. First, because our sample is derived from one hospital in Tokyo, the generalizability of our study result might be limited to some extent. Second, the Hb levels investigated in this study were categorized. Because of the low number of participants with a Hb level <7.0 g/dl, we were unable to perform multivariable analyses, however our sensitivity analyses confirmed a trend suggesting that the rate of successful breastfeeding would be lowest in Hb level <7.0 g/dl. Third, we did not collect information regarding iron supplementation. In our hospital, all pregnant women were advised to take iron supplements based on blood test results from both the second and third trimesters. Although we could not determine whether iron deficiency was successfully treated by iron supplementation, a very large proportion of our study participants had anemia levels <11.0 g/dl. Consequently, more active intervention by dieticians during pregnancy is required. Fourth, information regarding drinking and smoking statuses of the mother was collected only at the time of the first prenatal visit, and our results may be prone to non-differential misclassification bias.

## Conclusions

This study demonstrated a significant and negative impact of severe anemia, defined as a Hb level <9.0 g/dl, on successful EBF initiation. Such an inverse association was observed even with adjustment with amount of bleeding at delivery, suggesting that pro-anemic status of mothers in later trimester may also affect unsuccessful initiation of EBF.

## Abbreviations

EBF: Exclusive breastfeeding
GDM: Gestational diabetes mellitus
Hb: Hemoglobin
LBW: Low Birth Weight
PIH: Pregnancy-induced hypertension
